# Immunotherapy-associated cardiovascular toxicities: insights from preclinical and clinical studies

**DOI:** 10.3389/fonc.2024.1347140

**Published:** 2024-02-28

**Authors:** Youqian Kong, Xiaoyu Wang, Rui Qie

**Affiliations:** ^1^ Graduate School, Heilongjiang University of Chinese Medicine, Harbin, China; ^2^ First Affiliated Hospital, Heilongjiang University of Chinese Medicine, Harbin, China

**Keywords:** cardio-oncology, immune checkpoint inhibitors, immune-related adverse events, preclinical studies, clinical studies

## Abstract

Immune checkpoint inhibitors (ICIs) have become a widely accepted and effective treatment for various types of solid tumors. Recent studies suggest that cardiovascular immune-related adverse events (irAEs) specifically have an incidence rate ranging from 1.14% to more than 5%. Myocarditis is the most common observed cardiovascular irAE. Others include arrhythmias, pericardial diseases, vasculitis, and a condition resembling takotsubo cardiomyopathy. Programmed cell death-1 (PD-1)/programmed cell death ligand-1 (PD-L1) pathway, cytotoxic T-lymphocyte antigen-4 (CTLA-4) pathway, and the recently discovered lymphocyte-activation gene 3 (LAG-3) pathway, play a critical role in boosting the body’s natural immune response against cancer cells. While ICIs offer significant benefits in terms of augmenting immune function, they can also give rise to unwanted inflammatory side effects known as irAEs. The occurrence of irAEs can vary in severity, ranging from mild to severe, and can impact the overall clinical efficacy of these agents. This review aims to summarize the underlying mechanisms of cardiovascular irAE from both preclinical and clinical studies for a better understanding of cardiovascular irAE in clinical application.

## 1 Introduction

ICIs, also known as immune checkpoint inhibitors (ICIs), have revolutionized the field of cancer treatment, significantly improving the treatment options for a wide range of cancer types (1). The field of cancer treatment has witnessed a remarkable transformation over the past decade, attributed to the development of immunotherapy (2). This revolutionary method incorporates a variety of tactics that seek to strengthen or engage the immune system in recognizing and eradicating cancer cells. One of the pivotal advancements in this field is the introduction of ICIs therapy, which has completely transformed the landscape of cancer treatment. These checkpoint inhibitors have a specific focus on regulatory receptors found within the immune system, including PD-1, PD-L1, and CTLA-4. By binding to these inhibitory receptors, ICIs effectively stimulate the immune response, enabling it to recognize and attack cancer cells more efficiently (3, 4). The aforementioned monoclonal antibodies have received approval from the Food and Drug Administration (FDA) to treat a wide range of cancer types. These include melanoma, small-cell and non-small cell lung cancer (NSCLC), bladder cancer, basal cell carcinoma, Hodgkin’s disease, renal cell carcinoma, and any solid tumor with a microsatellite instability-high (MSI-H) or mismatch repair deficiency (dMMR) profile (5). The introduction of ICIs has revolutionized cancer treatment by achieving remarkable and long-lasting anti-tumor responses. In the past few years, ICIs have demonstrated remarkable achievements in the realm of cancer treatment. However, the usage of ICIs has resulted in a rise in irAEs, which are growing in frequency as the utilization of these drugs becomes more widespread (6).

The utilization of ICIs to augment the immune responses of the human body can lead to numerous types of toxicities associated with the immune system. Serious adverse effects, including inflammation of the colon, liver, lungs, thyroid gland, muscles, pituitary gland, and skin, can occur with the use of certain medications. However, these toxicities are usually temporary and can be successfully treated by administering glucocorticoid therapy (7). However, one particular toxicity that poses significant challenges to tumor treatment is cardiotoxicity caused by ICIs (8). This condition carries a high risk of death and seriously hinders the effectiveness of cancer treatment (9). The cardiotoxicity caused by ICIs includes various types of cardiac disorders, such as pericarditis, myocarditis, arrhythmias, acute coronary syndrome (ACS) and so on (10). Myocarditis stands out as the most prevalent and deadly among these conditions. The results of the endomyocardial biopsy (EMB) indicate that myocarditis induced by ICIs is characterized by the presence of lymphocytes infiltrating the heart tissue. It has been identified that the primary factor contributing to this process is the immune response mediated by T cells (11). In conclusion, with regards to the aforementioned matters, the objective of this review is to gain an in-depth understanding of the pathogenesis of this topic through the current research results, so as to reduce the incidence of sickness and death among cancer patients undergoing treatment with ICIs.

## 2 Mechanisms of immune checkpoint inhibitors

**Figure 1:**
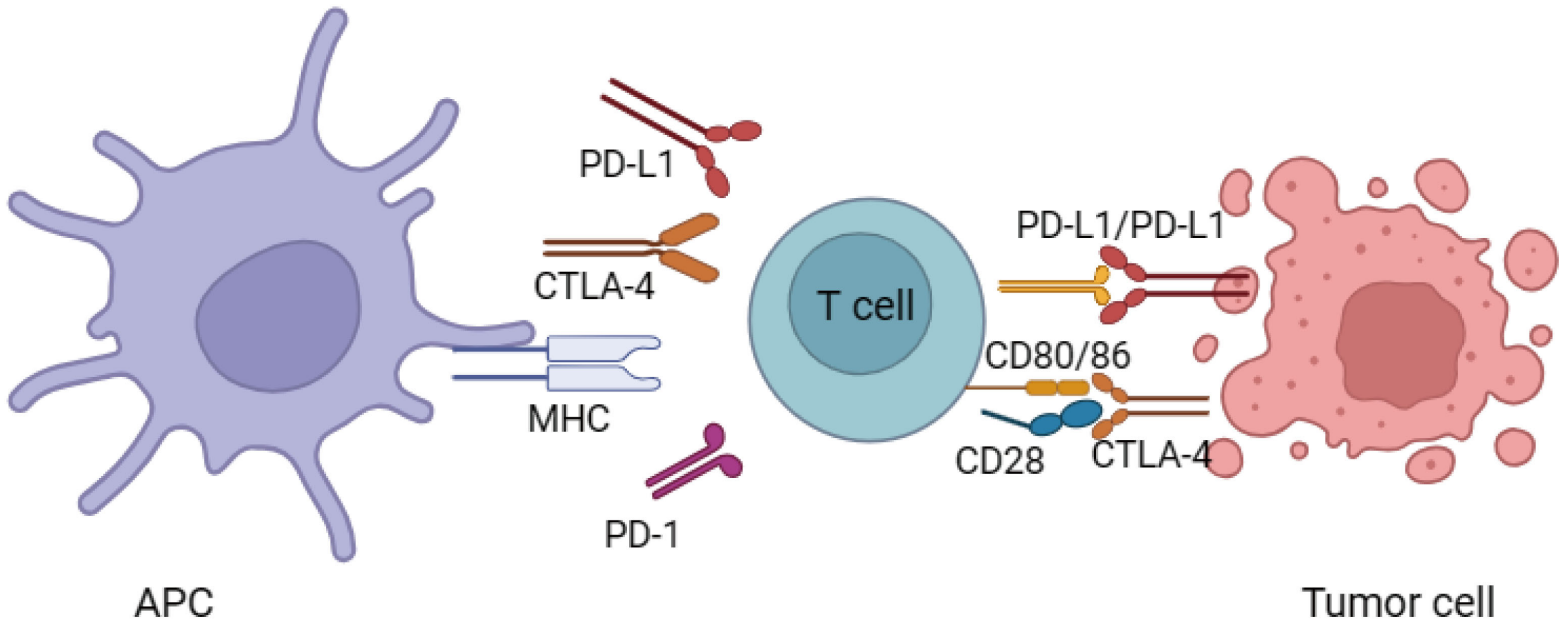
Mechanisms of immune checkpoint inhibitors.

These ICIs refer to specialized monoclonal antibodies that have the ability to boost the body’s immune response against cancer by interfering with immune system regulators known as down-regulators. These down-regulators include PD-1 and CTLA-4, as well as its associated ligand, PD-L1. By blocking these immune checkpoints, ICIs effectively unleash the activity of effector T-cells, thus facilitating a more robust anti-tumor response (1) (**Figure 1**). When T-cells are activated, the activation of CTLA-4 occurs. Activated T-cells and a specific subgroup of CD25+ CD4+ T-cells known as T-regulatory (T-reg) cells both exhibit the presence of CTLA-4 (12). Being a member of the immunoglobulin supergene family, CTLA-4 shares about 30% similarity with CD28. The affinity and avidity of its binding to CD80/86 are significantly greater than that of CD28. The binding of CTLA-4 to CD80/86 leads to the suppression of T-cell mediated immune responses. This occurs through the reduction of IL-2 and IL-2 receptor expression, ultimately resulting in a decrease in overall immune activity (13). Additionally, CTLA-4 can also influence immunity through its impact on T-reg cells (14).

There are distinct differences between the regulation of T cells through the PD-1-PD-L1 axis and that of CTLA-4. PD-1, a component of the immunoglobulin superfamily, becomes activated in peripheral T cells and B cells upon stimulation. Its primary role is to maintain peripheral tolerance (15). PD-1 engages with two ligands, namely PD-L1 and PD-L2, within the peripheral tissues. PD-L1 can be found in B cells, macrophages, T cells, and dendritic cells when they are in a resting state (16). The expression of PD-L2 is uncommon in quiescent immune cells; however, pro-inflammatory cytokines can stimulate its synthesis (16). The activation of both PD-1 and CTLA-4 pathways ultimately impacts the Akt signaling pathway; however, the specific pathways and outcomes of antibody inhibition vary (17). Akt, also known as protein kinase B (PKB), plays a pivotal role in regulating important cellular functions including metabolism, programmed cell death (apoptosis), and cell proliferation. The CD28 binding in T cells induces the activation of phosphatidylinositol 3-kinase (PI3K), which then associates with Akt, leading to its phosphorylation. While PD-1 signaling directly counteracts PI3K, CTLA-4 exerts its effects through the activation of PP2A, a phosphatase. Overall, these findings serve to emphasize the distinctions in the effects of anti-PD-1/PD-L1 and anti-CTLA-4 antibodies on T cells in relation to their activation stage, downstream pathways engaged, and site of action.

Currently, the FDA has granted approval to numerous ICIs for the management of diverse forms of cancer. These include ipilimumab (anti-CTLA-4), nivolumab, pembrolizumab, and cemiplimab (anti-PD-1), as well as avelumab, atezolizumab, and durvalumab (anti-PD-L1). With the ability to hinder the interactions between PD-L1 and PD-L2, anti-PD-1 agents hold promise. However, it has been noted that specific anti-PD-1 and anti-PD-L1 agents exhibit variations in terms of autoimmune toxicity. Numerous studies have unequivocally shown that the efficacy of PD-1 blockade and PD-L1 blockade in diminishing tumor growth is essentially identical (18).

## 3 Immune checkpoint inhibitors-induced Cardiotoxicity

**Figure 2:**
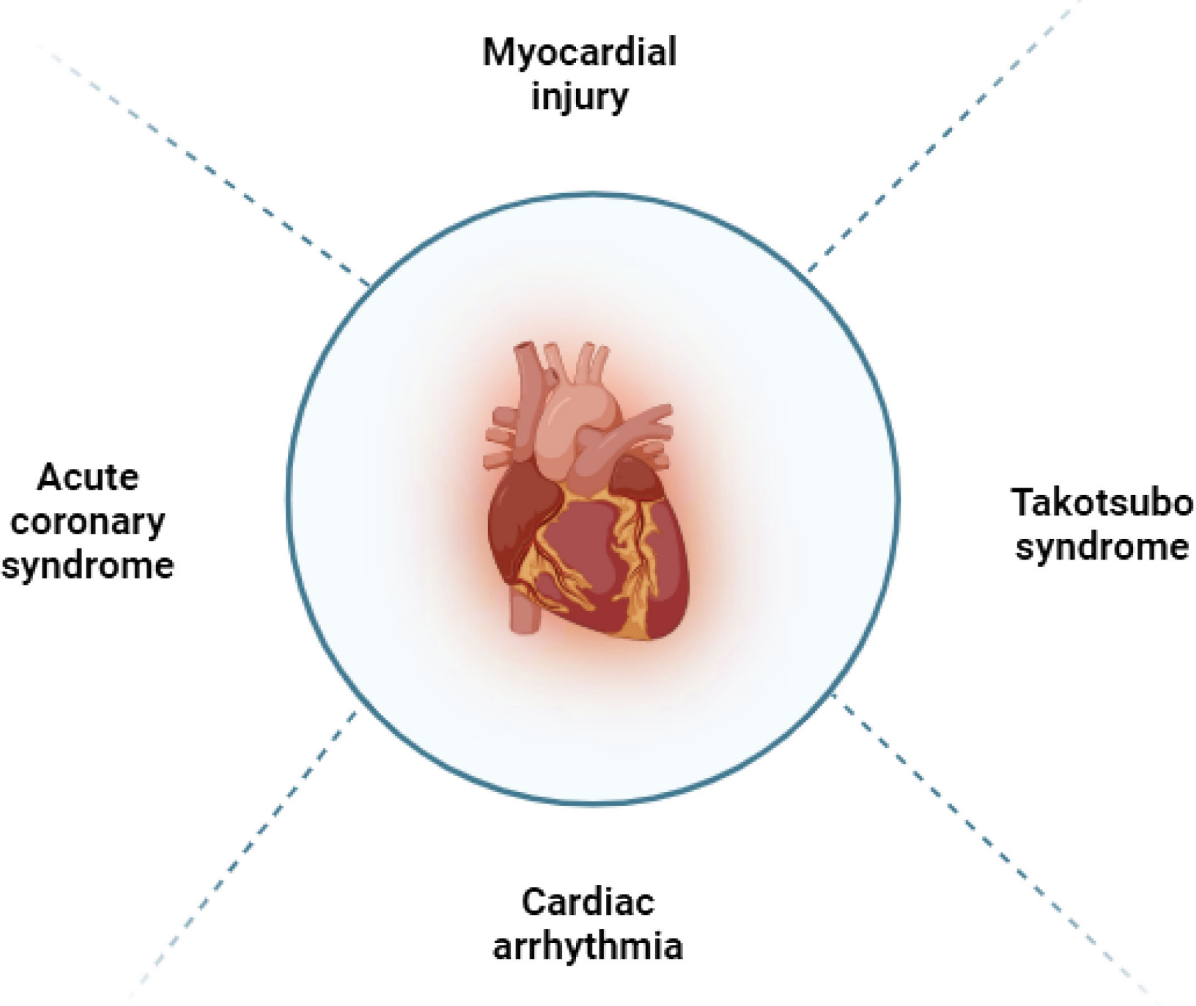
Types of cardiotoxicity induced by immune checkpoint inhibitors.

**Figure 3:**
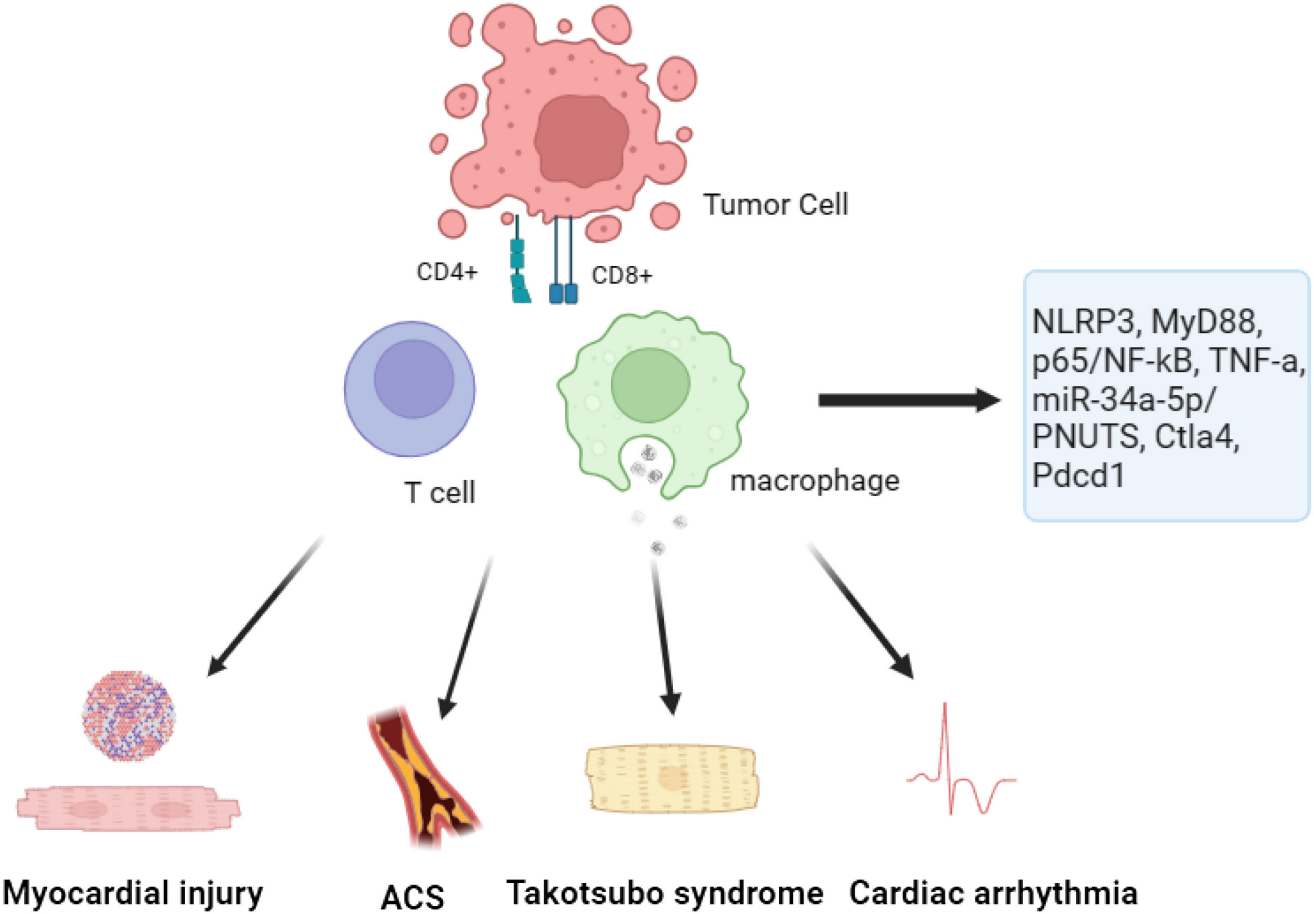
The mechanisms of immune checkpoint inhibitors-induced cardiotoxicity.

In general, the use of ICIs in patients may lead to irAEs, with a reported incidence rate of 60 to 80% (19) (**Figure 2**; **Table 1**). The PD-1 and CTLA-4 pathways are essential in regulating inflammatory cytokine and T cell-mediated immune activation in the heart (**Figure 3**). These pathways serve as immune checkpoints, actively suppressing excessive immune responses in the myocardium. Additional investigation is necessary to enhance our comprehension of the fundamental mechanisms and devise efficient approaches for the prevention and management.

**Table 1:** Cardiotoxicity induced by immune checkpoint inhibitors (ICIs).

	Disease classification	Cardiac irAEs	Immune Checkpoint Inhibitors	Target	Cancer	Diagnosis	Biomarkers	Ref
Clinical studies	Myocardial injury	Myocarditis	Nivolumab, Pembrolizumab, Ipilimumab, Atezolizumab, Durvalumab, Tremelimumab, Avelumab	CTLA-4, PD-1, PD-L1	Multiple cancers	Echocardiography, Electrocardiography	Troponin T, BNP or NT-proBNP, QRS duration, QTc interval, LVEF	(20)
		Myocarditis	pembrolizumab, nivolumab, or atezolizumab	PD-1, PD-L1	NSCLC	NA	NA	(21)
		Myocarditis	Avelumab, docetaxel	PD-L1	NSCLC	NA	NA	(22)
		Myocarditis	Nivolumab	PD-L1	gastric cancer	Echocardiography, Electrocardiography	hs-TnT, hs-TnI and CK-MB levels	(23)
		Myocarditis	nivolumab and ipilimumab	CTLA-4, PD-L1	Melanoma	Echocardiography, Electrocardiography	CK-MB, PR prolongation, QRS complexes, ST segment depression	(24)
		Heart Failure	Nivolumab, Pembrolizumab, Ipilimumab, Atezolizumab, Durvalumab, Tremelimumab, Avelumab	CTLA-4, PD-1, PD-L1		Echocardiography, Electrocardiography	LVEF, TnI, BNP or NT-proBNP	(25)
		Heart Failure	Nivolumab, Pembrolizumab, Atezolizumab, Durvalumab, ipilimumab	CTLA-4, PD-1, PD-L1	Lung cancer	Echocardiography, Electrocardiography	LVEF, TnI, BNP or NT-proBNP	(26)
	Disease classification	Cardiac irAEs	Immune Checkpoint Inhibitors	Target	Cancer	Diagnosis	Biomarkers	Ref
	ASC	Myocardial Infarction	Tremelimumab	CTLA-4	pleural mesothelioma; peritoneal mesothelioma	NA	NA	(27)
	TTS	TC	Ipilimumab	CTLA-4	metastatic melanoma	Echocardiography	ST elevations, LV outflow	(28)
	Cardiac arrhythmia	Atrial Fibrillation	Nivolumab	PD-1	metastatic adenocarcinoma of lung	Electrocardiography		(29)
		Arrhythmia	Pembrolizumab, Ipilimumab, Atezolizumab, Durvalumab, Tremelimumab, Avelumab	PD-1, PD-L1	advanced or metastatic solid tumors	Echocardiography, Electrocardiography	LVEF, TnI, MYO, CK, CK- MB, BNP, and LDH	(30)
		Arrhythmia	NA	CTLA-4, PD-1	Melanoma	Echocardiography, Electrocardiography	levels of autoantibodies and inflammatory cytokines	(31)
		Atrial Fibrillation	Nivolumab	PD-1	Melanoma	Echocardiography, Electrocardiography	LVEF, ESR and CRP	(32)
Pre-clinical studies	Inflammatory factor	Myocarditis	Nivolumab and Ipilimumab	CTLA-4 and PD-1	Cancer cell	Echocardiography	NLRP3, p65/NF-κB, NF-κBMyD88, and several interleukins	(33)
	Disease classification	Cardiac irAEs	Immune Checkpoint Inhibitors	Target	Cancer	Diagnosis	Biomarkers	Ref
		Myocarditis	Pembrolizumab and Ipilimumab	CTLA-4 and PD-1	NA	cardiac biomarkers	NT-proBNP, CK-MB, hs-TnT, NLR, NER, and CRP	(34)
	Immune factors	Atherosclerotic cardiovascular disease	Pembrolizumab and Nivolumab/Ipilimumab	CTLA-4 and PD-1	melanoma	Pathological staining of myocardial tissue	CD4+, CD3+, and CD8+	(35)
		Heart Failure	Nivolumab, Pembrolizumab, Ipilimumab, Atezolizumab, Durvalumab, Tremelimumab, Avelumab	CTLA-4, PD-1, PD-L1	Multiple cancers	NA	CD4+ and CD8+ T cells	(36)
	microRNAs	Heart Failure	NA	PD-1	NA	Echocardiography	miR-34a-5p	(37)
	Gene modification	Myocarditis	NA	PD-1	melanoma	Echocardiography	Ctla4 and Pdcd1	(38)
		Cardiac dysfunction	NA	PD-1	NA	Echocardiography	Dmd, Ass1, Chrm2, Nfkbia, Stat3, Gsk3b, Cxcl9, Fxyd2, and Ldb3	(39)

irAEs, immune-related adverse events; CTLA-4, cytotoxic T-lymphocyte antigen-4; PD-1, programmed cell death-1; PD-L1, programmed cell death ligand-1; NSCLC, small-cell and non-small cell lung cancer; LVID, left ventricular internal dimension; IL-17A, Interleukin 17A; LVEF, left ventricular ejection fraction; TnI, Troponin I; MYO, myoglobin; CK, creatine kinase; CK-MB, creatine kinase-MB; LDH, lactate dehydrogenase; BNP, brain natriuretic peptide; NT-proBNP, N-terminal pro brain natriuretic peptide.

NA, No available.

### 3.1 Epidimiology of irAEs from clinical studies

#### 3.1.1 Myocardial injury

According to a recent multicenter registry, major adverse cardiac events (MACE) were defined as a combination of cardiovascular death, cardiogenic shock, cardiac arrest, and hemodynamically significant complete heart block. The incidence of myocarditis was 1.14% and occurred at a median onset time of 34 days (interquartile interval 21 to 75 days) after beginning ICIs treatment (20). The ICIs-related myocarditis has proven to be the deadliest form of irAEs, with a fatality rate ranging from 27% to 46% (25, 40, 41). Therefore, the incidence of ICIs related myocarditis is low (1.14%), the mortality rate is high, the diagnosis is easy to be missed, and it is necessary to do a good job of disease education to patients. Typically occurring in the early phase of treatment, ICIs-related myocarditis can manifest as a severe and aggressive disease progression, leading to significantly impaired LV function, hemodynamic instability (42). A comprehensive examination was undertaken utilizing administrative claims information gathered from the OptumLabs^®^ Data Warehouse, a vast commercial insurance repository in the United States. The study aimed to identify patients with NSCLC who received a PD-L1 inhibitor between January 1, 2015 and December 31, 2017 (21). The results revealed that the incidence of any irAEs among patients with NSCLC who initiated a PD-L1 inhibitor was 52.5% after 12 months, based on a cohort of 3,164 individuals. Notably, the cumulative risks of irAEs showed an upward trend over time. Specifically, the risk of pericarditis stood at 1.7% after 9 months of treatment.

The JAVELIN Lung 200 trial was a significant international study conducted in multiple centers across 31 countries (22). This phase 3 trial involved 792 patients who were randomly assigned to receive either avelumab or docetaxel for their cancer treatment. Tragically, there were four treatment-related deaths in the avelumab group, with one specifically caused by autoimmune myocarditis. In response to these cases of myocarditis, Mahmood et al. established a comprehensive registry across eight medical sites (20). This registry included 35 patients who experienced myocarditis as a result of ICIs treatment. In order to obtain a more thorough comprehension, a comparison was made between these patients and a randomly selected group of 105 patients who underwent ICIs treatment but did not develop myocarditis. The analysis of the data revealed that the prevalence of myocarditis among ICIs-treated patients was found to be 1.14%. Additionally, it was observed that having elevated levels of troponin T resulted in a fourfold increased risk of experiencing MACE. These findings emphasize the importance of closely monitoring and managing irAEs in patients receiving PD-L1 inhibitors.

A recent study has shed light on the link between ICIs-induced myositis and asymptomatic myocarditis in cancer patients. The research encompassed an individual who was 79 years old and diagnosed with advanced stomach cancer that had spread to other parts of the body (23). This patient received treatment with nivolumab therapy. Although the patient exhibited mild elevation in cardiac markers, the presence of concurrent asymptomatic myocarditis was only identified after further investigation. Another research study revealed the unfortunate occurrence of two individuals diagnosed with melanoma who tragically experienced fatal myocarditis following their treatment with ipilimumab and nivolumab (24). The affected individuals did not just encounter a combination of myositis and rhabdomyolysis, but also showed early and resistant cardiac electrical irregularities. Moreover, their conditions were accompanied by significant infiltration of T-cells and macrophages, indicating the presence of myocarditis. According to pharmacovigilance data, it was found that myocarditis occurred in 0.27% of patients who received ipilimumab/nivolumab treatment. These cases serve as a reminder of the importance of thorough cardiac assessments in patients with ICIs-induced myositis, as early detection of concurrent myocarditis can significantly improve clinical outcomes.

To investigate further, this study conducted a comprehensive review of medical records from two cardio-oncology units between March 2015 and April 2017 (25). The study included a total of 30 patients who exhibited symptoms suggestive of ICIs-related cardiotoxicity. Among them, 12 patients were newly diagnosed in the two cardio-oncology units, while the remaining 24 patients had previously been reported in 10 different case series (43, 44). Information regarding these 24 patients was compiled from a recent review and two additional case reports. Diagnosis of cardiotoxicity occurred at a median of 65 days after the initiation of ICIs, typically following three infusions. Notably, the incidence of cardiotoxicity was higher following the first and third infusions. Clinical manifestations commonly observed included dyspnea, palpitations, and signs indicative of congestive HF. Furthermore, 79% of patients exhibited LV systolic dysfunction.

A thorough examination was carried out on a group of 252 lung cancer patients who were part of a study conducted at a single institution (26). These patients were treated with either immunotherapy utilizing ICIs or non-ICIs therapy. The objective of the study was to assess the occurrence of MACE, including cardiovascular mortality, non-fatal MI, non-fatal stroke, and hospitalization for HF. During a period of observation lasting for 6 months, 13.3% of patients who were administered ICIs experienced MACE, and the time interval from treatment to the occurrence of these events had a median duration of 51 days. Compared to that, the occurrence rate of MACE among patients not undergoing ICIs treatment was 10.3%, and the median interval before such events happened was 64 days. Significantly, a meticulous univariable Fine-Gray regression analysis, encompassing non-cardiovascular death as a competing risk, unveiled that the administration of ICIs did not exhibit any significant correlation with an escalated likelihood of MACE (45). According to additional multivariable regression analyses, it was found that patients who underwent treatment with ICIs and had higher levels of serum troponin I were more likely to be at an increased risk of encountering MACE. Moreover, individuals who underwent a combination of ICIs and either vascular endothelial growth factor inhibitors (VEGFIs) or tyrosine kinase inhibitors (TKIs) displayed an increased susceptibility to MACE (46). A significant proportion of cases involving MACE in patients receiving ICIs were primarily associated with hospitalizations due to HF.

#### 3.1.2 Acute coronary syndrome

In recent studies, there has been a notable rise in the occurrence of acute MI during clinical trials involving ICIs (47). The authors put forward the hypothesis that the use of ICIs therapy might contribute to the exacerbation of inflammation within atherosclerotic plaques, destabilization of pre-existing plaques, and facilitation of plaque rupture. There is conflicting evidence regarding the involvement of immune checkpoint signaling and its inhibition in the development of atherosclerosis.

The DETERMINE trial was an extensive investigation carried out at 105 research facilities across 19 nations, emphasizing strict adherence to its methodology (27). The study included individuals suffering from unresectable malignant mesothelioma in the pleural or peritoneal cavity who had previously undergone one or two systemic treatments for the advanced stage of the disease. A cumulative number of 571 individuals were assigned in a randomized manner to either be administered tremelimumab or a placebo, out of which 569 participants received the allocated treatment. During the treatment period, there were instances of unfortunate incidents resulting in fatalities among 36 out of 380 patients in the tremelimumab group and 12 out of 189 patients in the placebo group. These events included respiratory failure, myocardial infarction, and lung infection.

#### 3.1.3 Takotsubo syndrome

Takotsubo Syndrome (TTS) is a condition characterized by acute, reversible the LV dysfunction that can be mistaken for acute coronary syndrome. It is worth noting that a significant portion of TTS patients have a history of cancer, with prevalence rates reaching up to 29% (48). Despite its relatively low incidence, the mortality rate of TTS is high, as nearly 50% of myocarditis cases result in death. A recent study highlighted two cases of TTS that were newly identified as manifestations of cardiotoxicity associated with the administration of ICIs (49).

While previous studies on animals have indicated the possibility of ipilimumab, an immunomodulator, causing inflammation in the heart muscles, instances of clinically significant myocarditis as a result of this drug have been extremely uncommon. There was a documented instance involving a patient diagnosed with metastatic melanoma who underwent four regular doses of ipilimumab, and subsequently exhibited symptoms that resembled those of acute coronary syndrome (28). However, no culprit lesion was found during cardiac angiography, and echocardiography revealed a profile similar to takotsubo cardiomyopathy (TC), including apical ballooning, hyperdynamic basal wall motion, systolic anterior motion of the mitral valve, and severe LV outflow tract obstruction (50). Although not confirmed, this case raises the possibility that an autoimmune myocarditis induced by ipilimumab treatment could result in a TC-like syndrome.

#### 3.1.4 Cardiac arrhythmia

Nivolumab, a monoclonal antibody of the IgG4 isotype that belongs to a class of ICIs, has been developed to specifically bind to the PD-1 receptor. The objective of this therapy is to disturb the communication between PD-1 and its binding partners on malignant cells, consequently inhibiting T cell depletion in individuals suffering from cancer (29). However, while this mechanism can help unleash the immune system against cancer cells, it can also inadvertently lead to an autoimmune response against the body’s own tissues. This phenomenon is the primary cause of the adverse effects associated with nivolumab (51). In a particular instance, a 70-year-old man with advanced adenocarcinoma of the lungs underwent nivolumab therapy as a second-line treatment option (52). The recommended dose was 3 mg per kilogram administered every two weeks. Unfortunately, three days after receiving the second dose, he was admitted to the hospital due to increased shortness of breath and the sudden onset of AF with rapid ventricular response.

A thorough examination was carried out on a group of individuals diagnosed with cancer who received immunotherapy treatment at a specific healthcare facility from January, 2020, to February, 2022 (30). ICIs-associated cardiotoxicity was diagnosed by evaluating clinical symptoms, analyzing biochemical markers, and interpreting diagnostic imaging results. Among the 487 patients who were administered PD-1 or PD-L1 inhibitors, a total of 12 incidents of cardiotoxicity associated with ICIs were observed. All the individuals in question were diagnosed with advanced or metastatic solid tumors. The spectrum of cardiotoxicity varied in terms of severity, ranging from subclinical cardiac abnormalities presenting as asymptomatic elevation of troponin-I (TnI) levels to symptomatic cardiac abnormalities. Patients presenting with symptomatic cardiac abnormalities displayed a range of clinical presentations, such as accelerated heart rhythm (tachyarrhythmia) or slowed heart rhythm (bradyarrhythmia).

The research focused on patients diagnosed with lung cancer or malignant melanoma in Denmark from 2011 to 2017 (31). Multivariable Cox models were utilized for the examination of the connection between ICIs and cardiac events, with the calculation of absolute risks as a crucial component. The study encompassed a total of 25,573 lung cancer patients, out of which 743 received programmed cell death-1 inhibitor (PD-1i) treatment. The calculated incidence rate of cardiovascular events in these patients over the course of one year was found to be 9.7%. Out of the 13,568 individuals diagnosed with malignant melanoma, 145 individuals underwent PD-1i therapy, while another 212 received treatment with cytotoxic T-lymphocyte-associated protein-4 inhibitor (CTLA-4i) (53). The initial utilization of ICIs has been linked to the manifestation of cardiac events for the first time. Furthermore, this study revealed that the risk of arrhythmia was dependent on the duration of ICIs treatment.

There have been reports of a significant number of irAEs associated with the use of ipilimumab, a monoclonal antibody that specifically targets CTLA-4, and nivolumab, which targets PD-1. In July 2015, a 35-year-old individual with no previous medical issues discovered a swelling in the upper right area of their neck (32). Following a biopsy, it was confirmed that he had stage IV-M1a metastatic melanoma, with the primary lesion located on his scalp. The cancer had spread only to the cervical lymph nodes. In August 2015, he underwent surgical removal of the scalp lesion and cervical lymphadenectomy. Starting in October 2015, he received combination immunotherapy consisting of one dose of ipilimumab and nivolumab. However, in early December, he was diagnosed with AF and required electrical cardioversion to restore normal sinus rhythm.

### 3.2 Underlying mechanisms: insights from pre-clinical studies

#### 3.2.1 Inflammatory factor

The utilization of ICIs has also resulted in the emergence of adverse reactions. A study is to investigate the cytotoxic and pro-inflammatory effects of two specific ICIs, Nivolumab and Ipilimumab, as well as the underlying mechanisms and cytokine storm associated with this cardiac toxicity (33). In order to analyze the impacts, a combination of human heart muscle cells and immune cells were exposed to the drugs Nivolumab and Ipilimumab for research purposes. The researchers carried out experiments to evaluate the survival of cells and quantify the levels of NLRP3, leukotrienes, p65/NF-kB, and MyD88 expression. Furthermore, Ipilimumab was administered to C57 mice. Comprehensive assessments were conducted before and after the treatment using 2D-echocardiography, focusing on ejection fraction, fractional shortening, as well as radial and longitudinal strain. The levels of MyD88, NLRP3, p65/NF-kB, and 12 different cytokines were also analyzed in the cardiac tissue of the mice. The findings revealed that both Nivolumab and Ipilimumab exhibited remarkable anti-cancer capabilities when tested in co-cultures of lymphocytes with tumor or cardiac cells. Nevertheless, it was noted that these ICIs also resulted in notable cardiac toxicity. Compared to untreated cells, the expression of MyD88, NLRP3, and p65/NF-kB was elevated by both drugs. Nonetheless, the most notable adverse outcomes in terms of inflammation and cardiac toxicity were witnessed following the administration of Ipilimumab (54). After conducting further investigations on mice that received Ipilimumab treatment, it was observed that there was a notable decline in fractional shortening and radial strain. This suggests a deterioration in cardiac function as compared to the untreated mice (54). The observed results demonstrated a notable rise in the levels of MyD88, NLRP3, and various interleukins within the myocardium (55). In general, this research indicates that the medications Ipilimumab and Nivolumab demonstrate cytotoxic properties through their influence on the MyD88 and NLRP3/IL-1β pathways. These pathways ultimately lead to the development of a pro-inflammatory cytokine storm within cardiac tissue. These findings highlight the importance of closely monitoring and managing cardiotoxicity in patients receiving ICIs for cancer therapy.

The primary aim of this research was to examine the link between inflammatory indicators and the severity of ICIs-related cardiotoxicities (iRCs), as well as the prognosis of patients with iRCs (34). This retrospective study encompassed a period from January 2019 to December 2021 and involved the inclusion of 47 patients who had been diagnosed with iRCs. The patients were classified into two categories depending on the intensity of their iRCs: mild and severe. According to the research, it was discovered that the average duration from the initial administration of ICIs to the occurrence of iRCs stood at 35 days. At the initiation of iRCs, there was a marked elevation in cardiac biomarkers and inflammatory markers, surpassing their initial baseline values. Additionally, it was observed that the rate of NER at the onset of iRCs was significantly greater in patients with high-grade iRCs compared to those with low-grade iRCs. Through the classification of patients according to the median NER at the onset of iRC, it was revealed that an NER value equal to or greater than 184.33 was linked to advanced-stage iRCs and a mortality rate that was 36.3% higher when compared to the group with lower NER values. The findings indicated that patients who experience iRCs may have a notably increased NER during the initial onset of iRCs. Furthermore, there is a strong correlation between a higher NER value and the severity of iRC as well as an elevation in mortality rates. However, it is important to note that these results should be validated with larger datasets. In conclusion, this study highlights the potential utility of inflammatory markers, particularly the NER, in predicting the severity and prognosis of iRCs in patients undergoing ICIs therapy (56).

Emerging evidence suggests that ICIs may exacerbate pre-existing inflammatory conditions. This holds immense importance due to the crucial role that inflammation plays in the progression of atherosclerotic cardiovascular disease. To shed light on this topic, a recent research study delved into the possibility of short-term ICIs therapy exacerbating atherosclerosis (35). The researchers employed positron emission tomography-computed tomography, using the tracer (18)F-FDG, to identify inflammation in blood vessels and throughout the body caused by immune cells called macrophages in patients with melanoma who received treatment with pembrolizumab and nivolumab/ipilimumab (57). Furthermore, we investigated the potential inflammatory effects of immune checkpoint blockade by targeting CTLA-4 and PD-1 in atherosclerotic Ldlr^–/–^ mice. The study findings indicated that the administration of ICIs had no substantial effect on the uptake of (18)F-FDG in the major arteries, spleen, and bone marrow among individuals with melanoma. Furthermore, the administration of ICIs had no impact on the activation of myeloid cells found in the bloodstream and lymphoid organs of hyperlipidemic mice. However, noteworthy changes were observed in the adaptive immune response. While the size of the plaque did not alter, there was a noticeable change in the plaque’s composition, specifically shifting towards a more inflammatory phenotype driven by lymphoid cells. This shift was evident by a significant 2.7-fold increase in CD8+ T cells and an impressive 3.9-fold increase in the size of the necrotic core (58). Furthermore, there was an observed augmentation in the activation of endothelial cells, manifested by a 2.2-fold elevation in vascular cell adhesion molecule-1 and a 1.6-fold rise in intercellular adhesion molecule-1. This research offers significant findings on the impact of a treatment combination using anti-CTLA-4 and anti-PD-1 antibodies on vascular and systemic inflammation caused by myeloid cells in patients with melanoma, as well as in mice with hyperlipidemia (59). The findings indicate that such treatment does not significantly alter the aforementioned types of inflammation. It is important to highlight that the administration of short-term ICIs therapy in mice results in plaque inflammation mediated by T cells, which ultimately contributes to the progression of plaque.

In order to enhance the comprehension regarding the initial effects of PD-1 inhibition on cardiac well-being and to mitigate the occurrence of manifest cardiac ailments, an investigation was undertaken (60). The present study utilized a comprehensive strategy incorporating biochemical analysis and *in vivo* phenotyping to explore the distinct impacts of anti-PD-1 therapy on cardiac function (36). The study findings unveiled that PD-L1, a molecule located on the cells lining the heart’s blood vessels, acts as the primary communicator of immune interaction within the cardiac system. In an innovative mouse model for melanoma, researchers observed that treatment with anti-PD-1 therapy resulted in the infiltration of CD4+ and CD8+ T cells into the myocardium, with a notable increase in activation level for the latter. In addition, it was noted that the function of the left ventricle (LV) was compromised under the influence of pharmacological stress, as demonstrated through the use of pressure-volume catheterization. The dysregulation of myocardial metabolism had a consequential impact on both the proteome and lipidome, resulting in this impairment. Consistent with the results of the experiment, individuals suffering from metastatic melanoma who underwent anti-PD-1 therapy demonstrated diminished performance of their LV when subjected to stress. A significant finding emerged when researchers revealed that the inhibition of tumor necrosis factor alpha (TNF-α) could effectively maintain LV function, all while ensuring the anti-cancer effects of anti-PD-1 therapy remain uncompromised. This indicates that blocking TNF-a could potentially be a promising strategy to prevent the development of cardiotoxicity associated with ICIs (61). To summarize, the research revealed that anti-PD-1 therapy disrupts the balance of the immune system in the heart, leading to the early deterioration of myocardial functionality (62, 63). The discoveries presented in this study have significant implications for the prognosis of the increasing population of patients undergoing ICIs therapy. Through an in-depth examination of the TNF-a blockade, scientists may have discovered a potential approach to mitigate the development of cardiotoxicity linked to immunotherapy using ICIs.

#### 3.2.2 microRNAs

Exosomes play a crucial and important role in promoting cellular communication within the context of cardiac diseases. These small vesicles are responsible for transferring an assortment of biomolecules, with particular emphasis on microRNAs (miRs). One such miR is miR-34a-5p, known for its association with cardiac senescence. The purpose of this study was to investigate the potential correlation between the cardiovascular side effects of PD-1 inhibitors, commonly used as ICIs, and the transfer of exosomal miR-34a-5p, leading to cardiac senescence in a mouse model (37). The scientists made a remarkable finding whereby exosomes originating from macrophages that underwent PD-1 inhibitor treatment resulted in heightened levels of miR-34a-5p within cardiomyocytes. The increased expression of genes, combined with the process of aging in the heart, led to detrimental effects on cardiac health in mice. It was determined that miR-34a-5p acted as a transfer RNA within exosomes, contributing to cardiac senescence-related injuries. Additional experiments revealed that the pro-aging impacts triggered by exosomes from macrophages treated with PD-1 inhibitors in cardiomyocytes were mitigated when the expression of miR-34a-5p was inhibited in macrophages. Through TargetScan analysis and luciferase assays, it was revealed that miR-34a-5p targets the 3’-untranslated region of serine/threonine-protein phosphatase 1 regulatory subunit 10 (PNUTS) (64). The researchers concluded that exosomes originating from macrophages treated with PD-1 inhibitors possess pro-senescent properties by influencing the miR-34a-5p/PNUTS signaling pathway (65, 66). The discoveries provide potential focal points for alleviating heart injuries in cancer patients undergoing PD-1 inhibitor therapy.

#### 3.2.3 Gene modification

The application of ICIs in targeting PD-1/PD-L1 or CTLA-4 has brought about a groundbreaking transformation in cancer therapy. However, these therapies come with a downside-irAEs, among which myocarditis is a concerning one. In an innovative preclinical trial, researchers have successfully created a robust mouse model to explore the occurrence of myocarditis associated with ICIs. Through the incorporation of monoallelic loss of Ctla4 within the framework of complete genetic absence of Pdcd1, researchers accomplished the observation of untimely demise in approximately fifty percent of the mouse subjects (38). The cause of this untimely demise can be attributed to the invasion of T cells and macrophages into the heart, leading to critical disruptions in the electrocardiogram. The clinical and pathological characteristics observed in patients with ICIs-associated myocarditis closely mirror these findings. Furthermore, this research has unveiled a significant functional correlation between Ctla4 and Pdcd1, wherein their interaction efficacy is contingent upon the gene dosage levels. This finding provides a plausible mechanism for the increased occurrence of myocarditis in patients receiving combination ICIs therapy (24). Furthermore, it was demonstrated that intervention with CTLA-4-Ig (abatacept) effectively alleviated disease progression in the mouse model. Additionally, a case series of patients showed promising results in mitigating the aggressive course of ICIs-myocarditis with the administration of abatacept. In summary, this research not only establishes a reliable mouse model for studying ICIs-associated myocarditis but also highlights the significance of Ctla4 and Pdcd1 in the development of this adverse event. The potential therapeutic role of CTLA-4-Ig in mitigating the progression of ICIs-myocarditis is promising and warrants further investigation in clinical settings (67).

The research is dedicated to examining the influence of PD-1 blockade on heart function and uncovering the molecular pathways responsible for cardiotoxicity induced by ICIs (39). In order to conduct this study, C57BL6/J and BALB/c mice were given either an isotype control or an anti-PD-1 antibody. The researchers employed echocardiography as a means to assess the functioning of the heart and investigated alterations in cardiac gene expression through the utilization of bulk RNA sequencing. In order to evaluate the presence of inflammatory changes, qRT-PCR and immunohistochemistry techniques were employed on the cardiac tissue of the mice. Further studies were carried out in C57BL/6J mice by incorporating the use of anti-CD4 and anti-IL-17A antibodies, along with PD-1 blockade (68). The research results unveiled that the utilization of anti-PD-1 therapy in C57BL/6J mice led to the manifestation of cardiac dysfunction and LV enlargement, accompanied by a noticeable rise in nitrosative stress. Significantly, the restraining of IL-17A demonstrated remarkable efficacy in averting cardiac dysfunction induced by anti-PD-1 treatment in C57BL/6J mice. In addition, an analysis of alterations in gene expression profiles in the heart muscle of C57BL/6J and BALB/c mice showed distinctively regulated genes such as Dmd, Ass1, Chrm2, Nfkbia, Stat3, Gsk3b, Cxcl9, Fxyd2, and Ldb3, which play significant roles in cardiac morphology, signaling pathways, and inflammatory processes (69). In summary, the findings suggested that the inhibition of PD-1 could lead to impaired cardiac function in mice. Nevertheless, the effective prevention of ICIs-induced cardiac dysfunction can be achieved through the pharmacological inhibition of IL-17A (70). These discoveries shed light on the potential risks and mechanisms associated with immune checkpoint inhibitor usage in cancer therapy, specifically highlighting the importance of monitoring cardiac function and considering strategies to mitigate cardiotoxicity.

## 4 Discussion

Cardiac irAEs are not as common as irAEs in other organ systems, making them challenging to diagnose and manage (71). However, preclinical research has revealed that after an injury, human cardiomyocytes express PD-1 and PD-L1 (72). Studies conducted on mice lacking PD-1 and CTLA-4 have demonstrated the development of dilated cardiomyopathy and myocarditis (60, 73, 74). These animal experiments offer valuable insights into the underlying mechanisms of ICIs induced cardiotoxicity.

The pathogenesis of cardiotoxicity caused by ICIs is not fully understood. From a histological perspective, myocarditis associated with ICIs is distinguished by the infiltration of CD4+/CD8+ T cells, CD68+ macrophages, and a decrease in other immune cell populations within the myocardium (75). There is a notable rise in clonal cytotoxic Temra CD8+ cells, which exhibit distinct transcriptional alterations such as the upregulation of chemokines like CCL5, in both patients with ICIs-induced myocarditis and Pdcd1^-/-^ mice with myocarditis (76). The activation of these immune cells leads to the direct destruction of heart cells through cytotoxicity, as well as the release of proinflammatory cytokines, which contributes to the development of cardiac damage (77). Premature mortality occurs in mice that have been genetically modified to lack PD-1 and express CTLA-4, as they experience infiltration of T cells and macrophages into the myocardium. Additionally, these mice exhibit severe electrocardiographic abnormalities, which closely resemble the distinctive features of myocarditis associated with ICIs (78). Furthermore, dilated cardiomyopathy is observed in PD-1^−/−^ BALB/c mice due to the presence of auto-antibodies specifically attacking cardiac troponin (79). In a similar vein, the absence of PD-1 has been found to worsen cardiac damage in models involving CD8+ and CD4+ T cell-mediated myocarditis (80).

The absence of PD-1 in Murphy Roths Large (MRL) mice with autoimmune conditions led to the progression of severe myocarditis, a life-threatening condition marked by the infiltration of T cells and macrophages into the heart muscle. Additionally, the presence of positive cardiac specific antibodies was detected (81). In cancer patients undergoing ICIs therapy, different subtypes of B cells in their peripheral blood were found to be associated with treatment response (82). However, these B cell subtypes may also play a role in ICIs-related irAEs. Apart from antibodies specifically targeting the heart, researchers have also discovered autoantibodies that are generated as a result of ICIs therapy. These include antibodies against acetylcholine receptors, antibodies targeting striated muscle, and antibodies against mitochondria (83). Take, for instance, the detection of anti-striated muscle antibodies that have the potential to interact with antigens present in both cardiac and skeletal muscles. As a result, this interaction can trigger antibody-dependent cellular cytotoxicity (ADCC). In addition, these autoantibodies have the potential to trigger complement-dependent cytotoxicity (CDC), leading to the destruction of cells (84). The results of the study indicate that both cellular and humoral immune responses play a role in the development of cardiotoxicity associated with ICIs.

The precise mechanisms involved in recruiting immune cells to the myocardium remain largely elusive. The expression of immune checkpoints is not restricted to tumor cells alone. Instead, they are consistently expressed in various cell types such as antigen-presenting cells (APCs), endothelial cells, cardiomyocytes, and others. This characteristic makes them viable targets for ICIs (85). There exist numerous hypotheses regarding the mechanisms underlying cardiac irAEs associated with ICIs. There are several factors involved in this condition. These factors include the increased production of autoantibodies that attack the body’s own antigens, the activation of T cells throughout the body which target healthy heart tissues that bear similarities to tumor cells, and the occurrence of subtle or long-lasting inflammation caused by the infiltration of microbes (86). The coexistence of myocarditis and myositis can possibly be attributed to the presence of identical antigens in skeletal and cardiac muscle tissues, which are targeted by clonal T cells. There have been limited research conducted on the role of inflammatory cytokines in cardiac injury associated with ICIs. Several cytokines have been discovered, including interferon gamma, interleukin 1 beta (IL-1β), and chemokine (C-X-C motif) ligand 10 (CXCL10). The recruitment of neutrophils and macrophages to the heart is facilitated by the secretion of these cytokines and chemokines. In the context of HF, increased levels of these cytokines lead to the recruitment of T cells and other immune cells in the affected area, exacerbating the damage to the heart (87). There is an elevated likelihood of cardiac arrhythmias being triggered by certain cytokines, specifically IL-6 (36). Furthermore, certain cytokines have the potential to impact the process of excitation-contraction coupling, resulting in disrupted cardiac function due to their influence on calcium regulation (88). IL-1β, for instance, enhances the susceptibility to irregular heart rhythms by triggering the oxidation and phosphorylation of CaMK II in cardiac muscle cells (89).

In clinical practice, it is crucial to stratify the risk of MACE in patients who exhibit symptoms of ACS, and the critical time frame for emergency department MACE evaluation is set at 6 weeks, which equals to 45 days (90). When patients presented with chest pain and coronary syndrome, emergency department physicians stratified risk according to HEART scores: heart scores 0-3:1.6% were negative; heart score over 4 is positive. Negative patients are usually discharged from the emergency department for outpatient tests. The overall pooled prevalence of MACE was 15.4%. When using the HEART score, patients treated with ICIs cannot be compared to the study population. Therefore, the risk of developing MACE in ICIs-treated patients may be underestimated. If the attending physician is unaware of the potential irAE being treated by ICIs, the patient may be misdiagnosed. Therefore, when an ICIs-treated patient visits a hospital other than a cancer center, the primary physician must be aware of ICI’s treatment methods and potential side effects. Provide patients with information about ICIs treatment and provide 24/7 phone numbers to request consultations with cancer centers and oncologist cardiologists. And making old clinical results available to treating doctors outside the cancer center is more conducive to faster diagnosis and treatment. In addition, patients who will be treated by ICIs should undergo extensive cardiac examinations prior to ICIs treatment. If a patient experiences additional symptoms, such as chest pain, congestive heart failure (CHF), or arrhythmia, while undergoing immunotherapy with ICIs, the cardiologist can assess and compare the patient’s medical information prior to receiving ICIs with the new data obtained after the emergence of these symptoms.

## 5 Conclusion

ICIs have emerged as a promising therapeutic option across various cancer types, leading to a rapid expansion in their clinical applications. However, it is crucial to address the potential cardiotoxicities associated with ICIs, as they can give rise to severe and life-threatening complications. Timely recognition and treatment of these toxicities are essential to ensure optimal patient outcomes. To gain a mechanistic insight into irAEs and develop targeted interventions for mitigating such potentially fatal side effects, the utilization of pre-clinical animal models is indispensable. Moreover, these animal models can also contribute to our understanding of the pathogenesis of cardiovascular toxicities and aid in the development of predictive biomarkers. Efforts have already been invested in investigating the mechanisms underlying ICIs-related cardiotoxicity, and diagnostic approaches for early detection have been developed. However, a comprehensive understanding of how ICIs induce cardiac irAEs remains elusive, and there is still a lack of specific and noninvasive tests for diagnosing ICIs-related cardiac toxicity. Consequently, there is a pressing need for prospective and large-scale randomized controlled trials (RCTs) that assess the efficacy of different treatment strategies in managing ICIs-induced cardiac injury. To enhance the outcomes of cancer patients, future studies should be directed towards deepening our understanding of the mechanisms driving ICIs-related toxicity. These studies can facilitate the development of improved diagnostic, prognostic, and therapeutic approaches. By diligently pursuing such research endeavors, we can pave the way for better management of ICIs-related cardiotoxicity, ultimately benefiting cancer patients worldwide.

## Author contributions

YK: Data curation, Writing – original draft. XW: Formal Analysis, Methodology, Writing – original draft. RQ: Funding acquisition, Supervision, Writing – review & editing.
